# Climate change policies and health in Uganda: where are we headed?

**DOI:** 10.1093/inthealth/ihaf143

**Published:** 2025-12-12

**Authors:** Reagan Daniel Emoru, Leung Hei Lok Lancelot, Teddy Mbabazi, Charles Ssemugabo, Moffat Nyirenda

**Affiliations:** Department of Internal Medicine, Makerere University School of Medicine, P.O. Box 7072, Kampala, Uganda; Medical Research Council/Uganda Virus Research Institute and London School of Hygiene and Tropical Medicine Uganda Research Unit, Kampala, Uganda; Department of Rural Water Supply and Sanitation, Ministry of Water and Environment, Kampala, Uganda; Department of Disease Control and Environmental Health, Makerere University School of Public Health, P.O. Box 7072 Kampala, Uganda; Medical Research Council/Uganda Virus Research Institute and London School of Hygiene and Tropical Medicine Uganda Research Unit, Kampala, Uganda; London School of Hygiene and Tropical Medicine, London, UK

## Abstract

Over the past 2 decades, Uganda’s climate policies have steadily evolved, with several frameworks and legislative measures enacted. However, the explicit consideration of health impacts remains limited, with gaps in ministerial coordination, undefined health targets, insufficient funding and limited community engagement. This commentary examines current governmental strategies through a health lens, assessing how health impacts, mitigation and adaptation measures are addressed—and identifying opportunities to better integrate health into climate policies. Progress could be strengthened by improving ministerial coordination, setting explicit health targets, increasing funding, enhancing preparedness for extreme events such as floods, droughts and extreme heat, as well as climate-sensitive health outcomes through meaningful community engagement. We also identify priority research needs: developing practical health indicators, investigating the knowledge and training of health workers and assessing effective methods for community engagement and policy evaluation. Our findings highlight the need for a clear, actionable research agenda to guide policymakers and to enhance health system resilience and climate policy effectiveness in Uganda.

## Introduction

Climate change has gained increasing prominence in Uganda’s policy agenda, as seen by the growing number of climate-related policies and laws. However, the explicit consideration of health impacts within these policies remains limited. With Uganda expected to face significant climate-related health risks, there is an urgent need for research to inform policymakers on how best to integrate health considerations into climate policies and legislation.^1^ Uganda’s changing climate is already driving measurable increases in infectious disease burden. Malaria cases surge 30% in flood-affected communities compared with non-affected areas, with highland districts experiencing four-fold increases in prevalence as temperatures rise. Waterborne diseases follow similar patterns, with cholera outbreaks directly linked to flood events, including Lake Victoria outbreaks that negatively affect communities following contamination.^2^ This commentary provides an overview of the health dimensions of Uganda’s climate policies, based on a manual review of key policy documents identified through online literature searches with an analysis using a structured pre-piloted data extraction table to evaluate mitigation and adaptation measures, health-related content and stakeholder engagement mechanisms.

### Evolution of climate policies

Uganda’s first major national climate strategy, the National Adaptation Programmes of Action (NAPA), was published in 2007 and identified health as the fourth of eight priority areas.^3^ This initial step focused largely on community-level interventions, such as mosquito control and improving access to clean water.

In 2015, the National Climate Change Policy (NCCP) expanded Uganda’s climate framework, aligning with Vision 2040, the country’s long-term development strategy.^4^ Health once again was recognised as a priority sector, with infectious diseases highlighted as a climate-exacerbated threat. The policy also emphasized the importance of early-warning systems and preparedness for climate-related health risks.

The Climate Change Act of 2021 established legal frameworks for climate governance with formal reporting mechanisms.^5^ However, there was little explicit focus on health policies.

In 2022, Uganda aligned its climate policies with the Paris Agreement by updating the National Determined Contribution (NDC).^6^ The NDC introduced vulnerability and risk scores to each priority sector, including health. The contribution also highlighted water, sanitation and hygiene (WASH) targets for Uganda, setting concrete goals for adapting the water sector to climate threats.^6^

In August 2024, Uganda launched its Health National Adaptation Plan (H-NAP), the first of its kind in Africa, with the overarching goal of enhancing the resilience of the health sector against climate-related impacts by embedding adaptation into health strategies and planning. The H-NAP builds on the 2023 Climate Change Health Vulnerability and Adaptation Assessment , which surveyed 716 health facilities and revealed critical gaps in energy security, WASH management, workforce readiness and post-hazard recovery and structures its response across 10 components, from climate-smart leadership and workforce training to integrated risk monitoring and sustainable financing. The plan aims to safeguard continuous healthcare delivery amid floods, droughts and extreme heat.^7^

In addition to the aforementioned, Uganda also introduced a Green Growth Development Strategy in 2017 and the Uganda Country Planning Framework 2022–2027.^8,9^ Although these documents do not explicitly address health, their implementation could yield health co-benefits, e.g. through improved air quality and water resource management.

### Gaps in climate policies and health integration legislation

Uganda has made significant strides in climate adaptation over the last 2 decades. At the Seventy-eighth World Health Assembly in 2025, member states endorsed the draft Global Action Plan on climate change and health, providing a strategic framework for countries to adapt to climate-related health threats.^9^ Drawing on this global guidance and our policy review, we identify important gaps and opportunities for strengthening Uganda’s approach.

Many climate policies list health as a priority but fall short in articulating how health impacts will be systematically addressed and measured. Current policies lack climate-triggered health surveillance systems that could exploit observed 1- to 2-month lags between rainfall peaks and malaria outbreaks to enable warning and prepositioned interventions. Some health-relevant issues, such as WASH, are often categorised under other sectors, limiting opportunities for integrated monitoring of health outcomes. Currently, climate change and adaptation lie principally under the Ministry of Water and Environment. Increasing interministerial collaborations with the Ministry of Health on health-related climate issues may bring more health expertise into future responses. The government may also consult with public health experts to reduce the risk of overlooking health impacts of climate change.

Policymakers should establish clear, measurable health targets aligned with World Health Organization (WHO) recommendations. This is particularly important as climate adaptation efforts involve multiple ministries and require the support of local leaders. Clear health targets will improve the effectiveness of the proposed strategies and improve coordination both in central and local government. Specific and actionable health indicators—such as WASH coverage, rates of stockouts of essential medicines and malaria control metrics—could serve as key performance indicators (KPIs) to facilitate local monitoring. An interministerial taskforce should be created to strengthen coordination between the Ministries of Health and of Water and Environment. To support this, earmarked funds should be allocated to health-specific climate adaptation goals, ensuring sustainable financing, accountability and systematic evaluation of interventions.

Community engagement is equally critical to the success of national adaptation plans. Local governments could be made more accountable through clear KPI targets and routine monitoring. Strengthened surveillance of climate-related health issues, such as infectious disease outbreaks, would enable more agile responses. Increased training for health workers and local leaders on climate-related health risks should be prioritised. Local leaders can also help raise awareness through community-driven initiatives—e.g. water campaigns, mosquito net repair days and education on heat stress prevention. These community actions should also support the Long-Term Low-Emission Development Strategies (LT-LEDS) and Uganda’s carbon market mechanisms.^10^ International partners should continue to provide technical and financial support, aligned with the WHO resolution, to empower national and local actors.

### Opportunities for future research

Significant knowledge gaps remain, underscoring the need for research to inform effective policy and practice.

First, research should focus on developing practical health indicators that reflect progress in climate adaptation, as well as identifying climate metrics that best capture health impacts. Studies on feasibility, effectiveness and cost-effectiveness of early-warning systems for climate-related health risks—including those posed by droughts, floods and vector-borne diseases—are also needed.

Second, surveys and qualitative research should explore knowledge, capacity and training needs among health workers, government officials and community leaders. Understanding these gaps will help target funding and improve the impact of adaptation strategies.

Finally, research should assess methods for effective community engagement to raise climate-health awareness and mobilise local action. Understanding what interventions communities are most willing and able to adopt will help avoid investing in policies that fail to gain traction.

## Data Availability

Raw data for the findings of this commentary are available from the corresponding author upon request.
